# Investigation of a PID-Based Dynamic Illuminance Control System for Intelligent Neonatal Jaundice Phototherapy Using a Blue Light LED Array

**DOI:** 10.3390/s26020528

**Published:** 2026-01-13

**Authors:** Man Xie, Hongjie Zheng, Mei Liu, Xing Wen, Yile Fan, Bing-Yuh Lu

**Affiliations:** 1 School of Automation, Guangdong University of Petrochemical Technology, Maoming 525000, China; manxie666@gdupt.edu.cn (M.X.); 23034450228fyl@gdupt.edu.cn (Y.F.); 2 School of Engineering, Dali University, Dali 671003, China; zhenghongjie@stu.dali.edu.cn; 3 College of Mechanical and Electrical Engineering, Shihezi University, Shihezi 832000, China; wenxing@stu.shzu.edu.cn

**Keywords:** incubator design for photo therapy, feasibility of treatment, Arduino-based controller, blue light LED array

## Abstract

Newborns are unable to reliably express changes in their physical condition due to their physiological immaturity and limited capacity for communication; therefore, continuous and systematic monitoring during phototherapy is essential to ensure timely detection of adverse responses and maintenance of therapeutic safety. This study extends our prior work, which introduced an indirect method for measuring light intensity to improve precision in monitoring newborn skin illumination. Light-emitting diode (LED) phototherapy has attracted considerable attention as an effective treatment for neonatal jaundice (NNJ). This study introduces an three-dimensional configuration of blue LEDs. An Arduino Mega 2560 microcontroller with pulse-width modulation (PWM) technology was employed to independently regulate the intensity of LED strips, enabling precise control of light output. The strips were mounted on an arc-shaped structure that can be adjusted mechanically and electronically through pre-programmed instructions embedded in the microcontroller. The results demonstrate that blue light at a wavelength of 460 ± 10 nm aligns with the peak absorption spectrum of bilirubin, thereby optimizing the efficacy of phototherapy for NNJ. Both observed absorption peaks were within the therapeutically effective range. Computer simulations confirmed that stable output contours can be achieved using rapid electronic scanning with a PID control algorithm to dynamically adjust the duty cycle. Experimental data showed that LED radiation output was largely linear. This supports the use of linear control algorithms and confirms the platform’s feasibility for future research.

## 1. Introduction

Investigation on PID-Based Dynamic Control System for Intelligent Neonatal Jaundice Phototherapy Neonatal jaundice (NNJ), or hyperbilirubinemia, is a common condition in neonates, particularly in gynecological settings [1]. Prompt identification and appropriate management of NNJ are essential due to the risk of bilirubin-induced damage to the central nervous system. Even within physiological limits, elevated bilirubin levels can result in irreversible neuronal damage, manifesting as cerebral palsy, hearing loss, dental dysplasia, and upward gaze paralysis [2,3,4,5]. Accordingly, developing effective treatment strategies for NNJ is critically important. Phototherapy remains the standard clinical intervention for NNJ. Its efficacy is closely related to the wavelength of the light source, as bilirubin exhibits maximal absorption at 450–470 nm, corresponding to the blue-light spectrum. Therefore, blue-light sources are preferred in clinical NNJ phototherapy applications [6,7,8,9].

In recent years, light-emitting diodes (LEDs) and cold-light phototherapy have garnered considerable attention for treating patients with NNJ. Geido et al. [10] developed a cost-effective neonatal phototherapy system (biliLED) that employs LEDs with a dominant wavelength of 470 nm and a bandwidth of 35 nm. Their comparative analysis of various light sources—including fluorescent, halogen, pad/fiber, LED, mini-LED, and biliLED systems—has provided valuable insights for both medical and engineering professionals. Similarly, Kato et al. and Kuboi et al. [11,12] demonstrated that LEDs and devices with different spectral characteristics can achieve comparable therapeutic outcomes when the cyclobilirubin formation capacity is standardized by modifying the distance between the devices and patients. These studies highlighted additional advantages of LED-based phototherapy, including reduced power consumption and minimal thermal response.

However, ultraviolet (UV) radiation and blue-light sources are frequently discussed in conjunction due to their shared position at the high-energy end of the visible and ultraviolet spectrum, as well as their significant impacts on human health and technological applications [13,14,15,16]. During phototherapy, it is essential to ensure full eye protection for newborns, nurses, parents, and all relevant medical staff, with particular attention to protection against UV radiation. In addition, several researchers have investigated the potential ocular hazards associated with blue light exposure, particularly in the wavelength range of 415–455 nm, which can penetrate the cornea and lens to reach the retina. Ouyang et al. [17] reported that higher energy levels can lead to irreversible photochemical damage, contributing to conditions such as dry eye syndrome, cataracts, and age-related macular degeneration. Moreover, concerns have been raised regarding the broader physiological effects of blue light, including its impact on circadian rhythms [18], the immune system [19], hormones [20], and daily activity. Therefore, medical personnel operating phototherapy devices may be at risk of adverse effects from prolonged exposure to blue light. Implementing effective protective measures is essential to mitigate these risks, ensuring the safety of healthcare staff and the effective operation of phototherapy equipment.

The correlation between radiation intensity and the distance from the blue-light source to the skin of newborns undergoing therapy has been extensively investigated. Choi et al. [21] investigated the control of blue-light laminates using an organic LED (OLED) as a voltage source. Sebbe et al. [22] characterized an optical device featuring an array of blue LEDs for treating NNJ and highlighted the non-uniform distribution of luminance generated by the array. Subsequently, Colindres et al. [23] conducted a randomized controlled trial using low-cost LEDs to modify the distribution of blue-light radiation for treating neonatal hyperbilirubinemia. Hashim et al. [24] employed an Arduino Uno and computer vision system to regulate LED radiation distribution, thereby ensuring real-time safety during neonatal treatments. Similarly, Fadilla et al. [25] investigated a multi-functional infant incubator monitoring system that incorporated phototherapy and an ESP32-based mechanical swing for uniform light radiation. Building on these foundational studies, the proposed method introduces a hybrid approach that combines electrically controlled and mechanically scanned LED strips. By employing pulse-width modulation (PWM) with time-variant control, this system effectively addresses the challenge of non-uniform light distribution, enhancing both the safety and effectiveness of neonatal phototherapy.

The primary objective of the proposed design is to advance blue-light phototherapy incubators for NNJ treatment by minimizing blue-light leakage and enhancing safety for both newborns and medical personnel. Key protective features include an opaque enclosure, real-time monitoring of the incubator interior, and programmable digital logic for additional safety assurance. The design and implementation of the proposed phototherapy system focus on reducing unnecessary blue-light exposure while leveraging technological innovations to deliver safe and compassionate care for newborns undergoing phototherapy.

Newborns, as vulnerable and developing individuals with limited sensory expression, are unable to communicate their discomfort effectively. Therefore, continuous and meticulous monitoring is essential during phototherapy—a critical process in which all variations in light exposure and physiological responses must be closely observed to ensure timely identification of potential risks and the maintenance of patient safety. This study builds on our previous research [26], which introduced an innovative indirect method for measuring light intensity, enabling more precise and consistent monitoring of illumination on newborn skin to support improved clinical care. By implementing electronic LED scanning, the system allows dynamic adjustments to radiation patterns, thereby supporting a wider range of treatment configurations tailored to diverse clinical presentations of NNJ. However, the scanning process also raises concerns regarding the stability of illuminance, which necessitates further investigation. An indirect measurement approach was developed to evaluate illumination levels on neonatal skin and thus address the abovementioned challenge, facilitating real-time control of light intensity and enhancing the overall safety of phototherapy. This study also presents and discusses the differences in illumination performance between open- and closed-loop control systems. The remainder of this study is organized as follows: Section 2 provides a brief introduction to the PID controller and the experimental setup; Section 3 presents the results of illumination measurements and experimental tests; Section 4 and Section 5 outline the discussion and conclusions, respectively.

## 2. Materials and Methods

**Figure 1:**
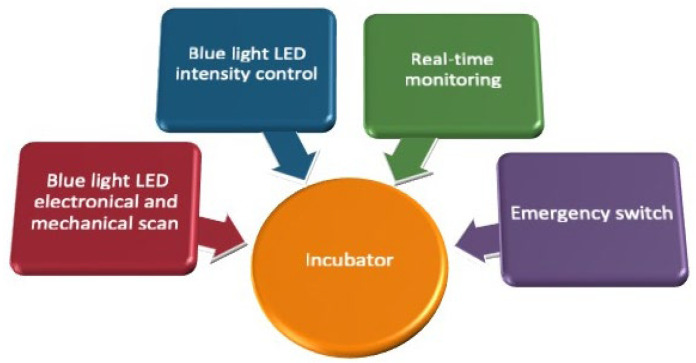
Functional block diagram of the blue-light phototherapy incubator. The average voltage delivered to the LEDs is directly dependent on the duty cycle, and the average values are adjusted by varying w. Therefore, the power intensity of radiation (*P*) can be controlled linearly, as described below. where $P_{on}$ denotes the power intensity during the “ON” state of the LED.

The system integration of the components of the blue-light phototherapy incubator is shown in Figure 1. An Arduino Mega 2560 microcontroller (Arduno^TM^, Monza, Italy) utilizing PWM technology was employed to regulate the intensity of the individual LED strips, thereby enabling precise control of light output. The blue LED strips were mounted on an arc-shaped frame, which could be moved mechanically using a pair of conveyor belts (mechanical scanning) or electronically via microcontroller programming (electronic scanning). Two light meters were employed to determine the correction factor (CF) for each scanning mode of the LED array and to provide real-time monitoring of illumination on the skin surface of newborns. For enhanced safety, an emergency stop button was installed on the rear side of the phototherapy incubator, allowing immediate disconnection of all power sources in the event of an emergency.

### 2.1. PWM

PWM technology was employed to regulate the intensity of individual LED strips, enabling accurate control of light output. PWM operates by generating a signal that alternates between the “ON” and “OFF” states during specific periods *(T_on_* and *T_off_*, respectively). The duration during which the signal remains “ON” is referred to as the duty cycle (w). A duty cycle of 100% indicates that the signal is continuously “ON.” The duty cycle can be calculated as follows:

(1) $$w=\frac{T_{on}}{T_{on}+T_{off}}\cdot 100\%,$$

(2) $$P=wP_{on},$$

### 2.2. Duty Cycle of PWM Versus Illumination of Turned-ON LEDs as a Function of Column with Electronic Scanning

**Figure 2:**
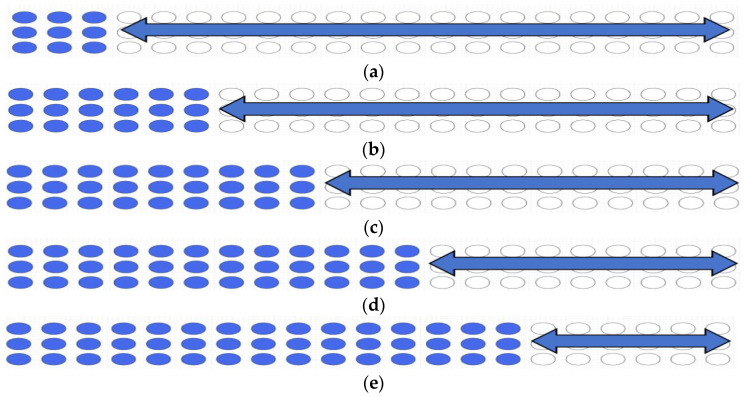
Configurations of the turned-ON LEDs as a function of the column with electronic scanning for different numbers of turned-ON rows (Nc): (**a**) Nc = 9; (**b**) Nc = 18; (**c**) Nc = 27; (**d**) Nc = 36; (**e**) Nc = 45. Turned-ON and turned-OFF LEDs are represented by blue and white circle, respectively.

Following previous studies [26,27], illumination measurements were conducted at two locations: the center of the phototherapy compartment (pc) and the central edge of the treatment compartment near the newborn’s feet (pe). The arc-shaped LED arrays, comprising 3 rows and 20 columns, were designed to allow column-by-column activation. The configuration of the LEDs turned ON through electronic scanning for different numbers of turned-ON rows (*N*_c_) is shown in Figure 2, for *N*_c_ values of 9, 18, 27, 36, and 45. Turned-ON and turned-OFF LEDs are represented by blue and white columns, respectively. Assuming that theoretical linear combinations are maintained, the following equations can be applied:

(3) $${\bar{I}}_{p_{c}}\left(N_{c}\right)=\frac{\int_{0}^{T}N_{c}I_{p_{c}}\left(t\right)dt}{T},$$

(4) $${\bar{I}}_{p_{e}}\left(N_{c}\right)=\frac{\int_{0}^{T}N_{c}I_{p_{e}}\left(t\right)dt}{T},$$

where ${\bar{I}}_{p_{c}}\left(N_{c}\right)$ and ${\bar{I}}_{p_{e}}\left(N_{c}\right)$ denote the average scanning illumination at *p*_c_ and *p*_e_, respectively. $I_{p_{c}}\left(t\right)$ and $I_{p_{e}}$ denote the instantaneous illuminations at these two points. The switching period of a step of LED movement (*T*) is defined as 2 s. Accordingly, CF is expressed as follows:

(5) $$CF\left(N_{c}\right)=\frac{{\bar{I}}_{p_{c}}\left(N_{c}\right)}{{\bar{I}}_{p_{e}}\left(N_{c}\right)}$$

### 2.3. Closed-Loop Control: PID Controller

Accurate measurement of illumination on the newborn, achieved through the indirect measurement method, facilitates the straightforward implementation of closed-loop control algorithms. Compared with previous studies utilizing open-loop control, our design enhances both the accuracy and safety of NNJ phototherapy. For example, a simple PID controller can be dedicated to the NNJ phot treatment as follows:

(6) $$I_{c}\left(t\right)=k_{P}e\left(t\right)+k_{I}\int e\left(t\right)dt+k_{D}\frac{de\left(t\right)}{dt}$$

where $e\left(t\right)$= $I_{sp}$ − $I_{c}\left(t\right)$. The desired illumination (set point) is denoted as $I_{sp}$, while the proportional, integral, and derivative constants are represented as $k_{P}$, $k_{I}$, and $k_{D}$, respectively. In the experiments conducted in this study, the parameter settings were $I_{sp}$ = 100 lx, $k_{P}$ = 0.9, $k_{I}$ = 0.1, and $k_{D}$ = 0.001.

### 2.4. Equipment

#### 2.4.1. Spectroradiometer

The HPCS-320 spectroradiometer (Rainbow^TM^, Hangzhou, China) is equipped with a high-speed AMR processor and a high-precision electronic charge-coupled device optical sensor. Developed based on the cross-asymmetric Czerny–Turner spectrometry principle, the device features a compact design, stable performance, high accuracy, cost-effectiveness, and portability. The device specifies a wavelength range of 380–780 nm, with a wavelength accuracy of 0.5 nm, an illuminance measurement range of 10 lx to 200 klx, and an illuminance accuracy of ±4%. The spectroradiometer is equipped with a 3.5-inch high-resolution capacitive touch screen, providing an intuitive, smartphone-like user interface. This configuration allows real-time sampling and measurement of light-source parameters at any location. When connected to a dedicated PC software (Arduino IDE), the system enables efficient analysis and processing of test data.

#### 2.4.2. Illuminance Meter

The Sanliang-PP730 illuminance meter (Sanliag^TM^, Tokyo, Japan) provides high measurement accuracy, continuous data acquisition, and raw data export capabilities. During experiments, data were transmitted in real time to the host computer via Bluetooth communication. The host computer then visualized the collected data as line charts, facilitating more effective experimental analysis.

#### 2.4.3. Digital Illumination Sensing Module

The GY-30 digital illumination sensing module employs the ROHM BH1750FVI chip (ROHM^TM^, Kyoto, Japan) and operates at a supply voltage of 3–5 V, providing an illuminance measurement range of 0–65,535 lx, with a resolution of 1 lx. The sensor features an integrated 16-bit analog-to-digital converter, enabling direct digital output without requiring calibration or complex computational procedures. Its spectral sensitivity closely matches human visual perception, supporting illuminance measurements as low as 1 lx. Data communication was implemented using a standard NXP I^2^C interface (Philips Semiconductors, Eindhoven, The Netherlands), and the integrated level-shifting circuitry of the module enables direct interfacing with 5 V microcontrollers. The sampling rate of the module was set to 2 Hz.

### 2.5. Experiment

#### 2.5.1. Open-Loop Control

**Figure 3:**
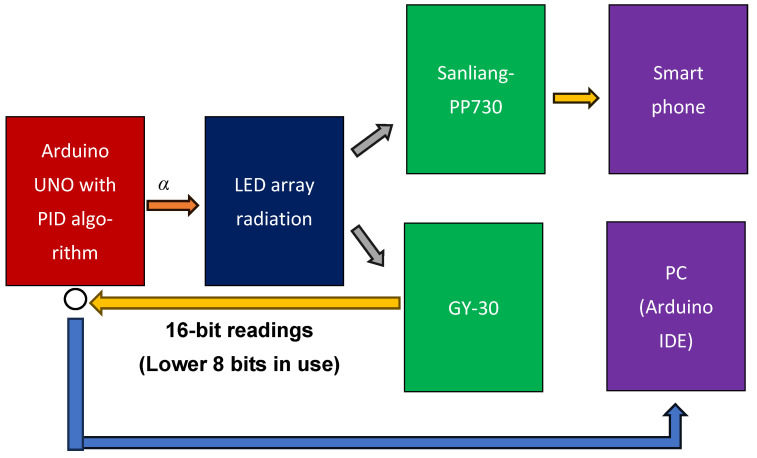
Open-loop control. The white circle means a data conversion in Arduino from 8-bit parallel port into a serial port connected with PC to display the readings of GY-30.

Open-loop control is a fundamental type of control in which the output does not influence the input and no feedback mechanism is available to correct errors or compensate for external disturbances. Open-loop control systems operate strictly according to predefined instructions, irrespective of the actual outcomes. In this case, the open-loop control indicates the initial input PWM setting (α) that produced a measured light intensity of 100 lx when the radiating LEDs were positioned at the center of the array, as shown in Figure 3, where α is a constant. The positions of Sanliang-PP730 and GY-30 are pc and pe, respectively. Their readings are denoted as OL@pc and OL@pe, respectively. The OL@pc values were transmitted to a smartphone (OPPOTM, A77 5G, Shenzhen, China) via Bluetooth communication, while OL@pe readings were sent to an Arduino connected to a notebook personal computer (ASUSTM, LAPTOP-H8C11FNJ, Taipei, Taiwan). These readings were displayed with the Arduino integrated development environment (IDE) serial port window. In Figure 3, the white circle means a data conversion in Arduino from 8-bit parallel port into a serial port connected with PC to display the readings of GY-30.

#### 2.5.2. Closed-Loop PID Control

**Figure 4:**
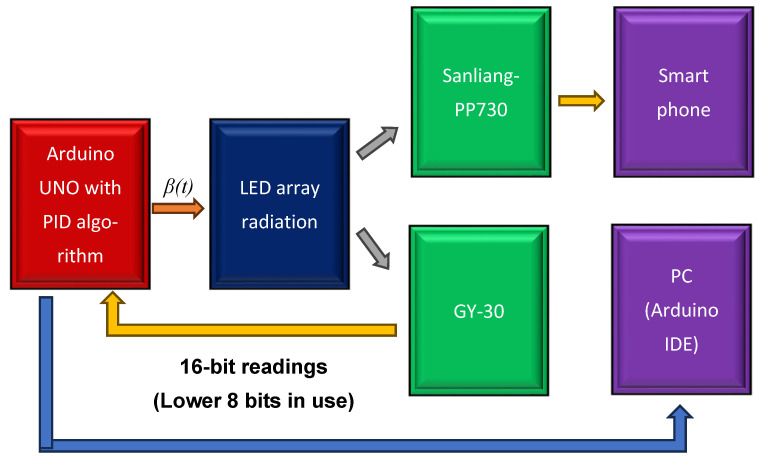
PID control.

In this experiment, the GY-30 sensor readings were used by Arduino UNO to implement PID feedback control, as shown in Figure 4. Only the lower 8 bits of the 16-bit GY-30 output were processed by the Arduino UNO, which executed the embedded PID control algorithm. Parameter conversion from the results of this experiment to Equation (6) is expressed as follows:

(7) $$e\left(t\right)=\alpha -{CF}_{N}\left(x\right)\cdot (PID@pe),$$

where x represents the PWM value, and ${CF}_{N}\left(x\right)$ is listed in Appendix A. Notably, the measurement resolution was 1 lx. The experimental PID controller can be mathematically expressed as follows:

(8) $$\beta \left(t\right)=k_{P}e\left(t\right)+k_{I}\int e\left(t\right)dt+k_{D}\frac{de\left(t\right)}{dt}.$$

Thus, $I_{c}\left(t\right)=\beta \left(t\right).$ The functional block diagram of this configuration is shown in Figure 4.

#### 2.5.3. Tuning Process of PID Control

The goal is to find the optimal PID gains (*k_P_*, *k_I_*, *k_D_*) for desired closed-loop performance—fast response, minimal overshoot, zero steady-state error, and good disturbance rejection—while maintaining robustness. The process is iterative and includes:

1. Initialization and Pre-Tuning
  (1) Understand the system: Identify key dynamics such as time constants, delays, nonlinearities, and constraints.
  (2) Define specifications: Set clear targets (e.g., <5% overshoot, <10 s settling time).
  (3) Use P-only control: Set *k_I_* = 0, *k_D_* = 0. Increase *k_P_* until sustained oscillations occur. Record the ultimate gain (Ku) and oscillation period (Pu)—the basis for Ziegler-Nichols closed-loop tuning.
2. Tuning Methods
  (1) *k_P_*: Increase to reduce rise time and steady-state error, but increases overshoot and oscillations.
  (2) *k_I_*: Add to eliminate steady-state error, but increases overshoot and settling time; slightly reduce Kp.
  (3) *k_D_*: Add to reduce overshoot and oscillations, improve stability. Highly sensitive to noise.
3. Implementation and Fine-Tuning
  (1) Apply gains: Start with conservative values (e.g., 50–80% of calculated gains).
  (2) Test performance: Run step response tests for setpoint tracking and disturbance rejection.
  (3) Adjust iteratively: Tune gains to balance performance. If overshoot is high, reduce *k_P_* or increase *k_D_*.
  (4) Handle real-world effects: Add a filter to the derivative term (time constant N ≈ 5–20) to reduce high-frequency noise.

In our experience, the performance of P-control already made the error be reduced. Then, we fine-tuned k_i_ and k_d_ for all the experiment, i.e., *k_P_* = 0.9, *k_I_* = 0.1, *k_D_* = 0.001.

## 3. Results

**Figure 5:**
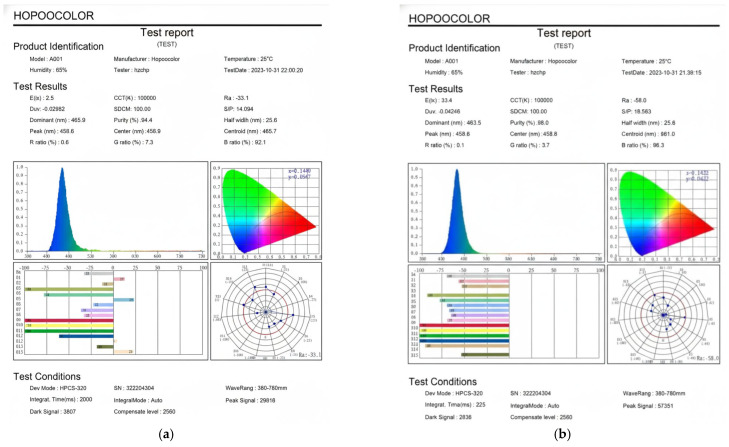
Reports of blue light measurement. The LEDs on the inner side of the cover were all turned ON with a duty cycle of 100%; the blue illuminations were then equal to (**a**) 0.366 W/m^2^ or 2.5 Lux (covered) and (**b**) 4.89 W/m^2^ or 33.4 Lux (uncovered) when measured outside of the phototherapy incubator.

All LEDs on the inner side of the cover operated under a 100% duty cycle. Under these conditions, the measured blue light intensities were 0.366 W/m^2^ (covered) and 4.89 W/m^2^ (uncovered), corresponding to 2.5 lx (covered) and 33.4 lx (uncovered), as measured outside the phototherapy incubator using the HPCS-320 spectroradiometer (Hopoocolor Tech., Hangzhou, China). The results are shown in Figure 5. Moreover, spectral peaks were observed at 465.9 nm and 463.5 nm, respectively. The distance between the phototherapy incubator and the measurement point was approximately 1 m. As highlighted in the introduction, the therapeutic efficacy of NJJ phototherapy is strongly dependent on the emission wavelength of the light source. Blue light in the range of 460 ± 10 nm closely corresponds to the peak absorption spectrum of bilirubin and is therefore recommended for clinical phototherapy in NNJ treatment [6,7,8,9]. Both measured peaks in this study fall within this effective therapeutic range. However, O’hagan et al. [28] reported that wavelengths between 430 and 470 nm pose the greatest risk of ocular damage, according to the blue-hazard action spectrum.

**Figure 6:**
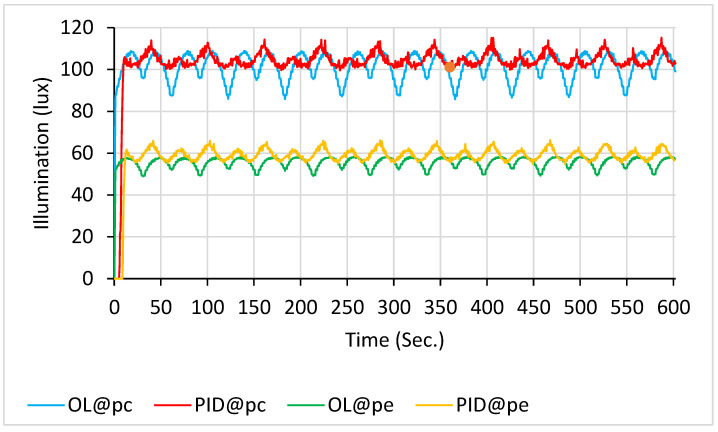
Illuminance with 9 LEDs scanning: PID control (at p_c_ in red line and at p_e_ in orange line) vs. open-loop control (at p_c_ in blue line and at p_e_ in green line).

This range overlaps substantially with that used in blue-light phototherapy, necessitating protective measures. In this study, the incubator cover served as a light radiation shield, significantly reducing blue-light leakage when closed and thereby enhancing safety for medical staff, parents, and other personnel. As shown in Figure 6, the open-loop system exhibited pronounced fluctuations when nine LEDs were used in scanning mode. The mean illuminance (M) and standard deviations (SD) over a 10 min period were as follows: 101.5 ± 9.2 lx for the blue line (illuminance at p_c_ under open-loop control, OL@p_c_); 103.9 ± 6.6 lx for the red line (illuminance at p_c_ under PID control, PID@p_c_); 55.8 ± 3.5 lx for the green line (illuminance at p_e_ under open-loop control, OL@p_e_); and 59.0 ± 2.9 lx for the orange line (illuminance at p_e_ under open-loop control, PID@p_e_).

**Figure 7:**
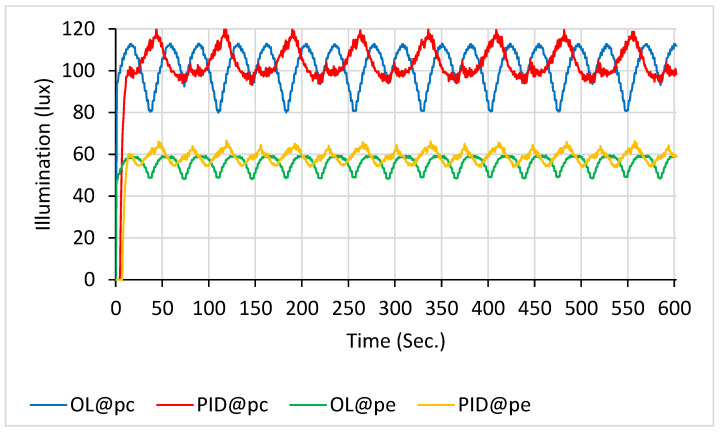
Illuminance with 18 LEDs scanning: PID control (at p_c_ in red line and at p_e_ in orange line) vs. open-loop control (at p_c_ in blue line and at p_e_ in green line).

With 18 LEDs in scanning mode (Figure 7), the open-loop system again demonstrated substantially greater fluctuations than other configurations. Over a 10 min interval, the average illumination values were as follows: 101.6 ± 6.1 lx for the blue line (OL@p_c_), 104.7 ± 3.1 lx for the red line (PID@p_c_), 55.8 ± 2.4 lx for the green line (OL@p_e_), and 59.4 ± 2.5 lx for the orange line (PID@p_e_).

**Figure 8:**
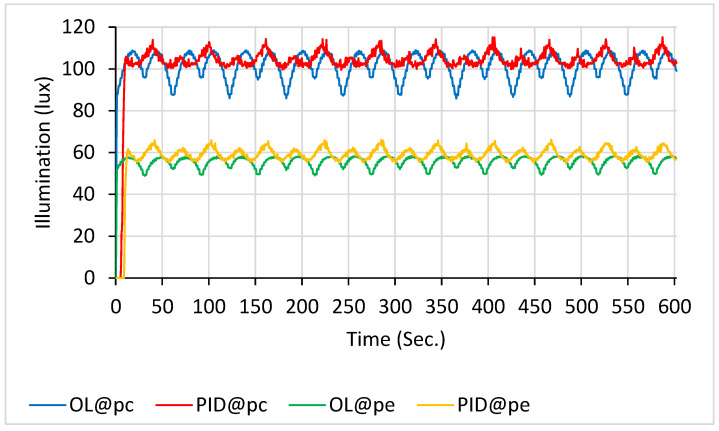
Illuminance with 27 LEDs scanning: PID control (at p_c_ in red line and at p_e_ in orange line) vs. open-loop control (at p_c_ in blue line and at p_e_ in green line).

The illuminance of the OL and PID controls at p_c_ and p_e_ in the 27-LED scanning configuration is shown in Figure 8. The average values within a 10 min duration were as follows: 99.9 ± 3.4 lx for the blue line (OL@p_c_), 101.2 ± 3.0 lx for the red line (PID@p_c_), 54.2 ± 1.3 lx for the green line (OL@p_e_), and 56.1 ± 1.9 lx for the orange line (PID@p_e_).

**Figure 9:**
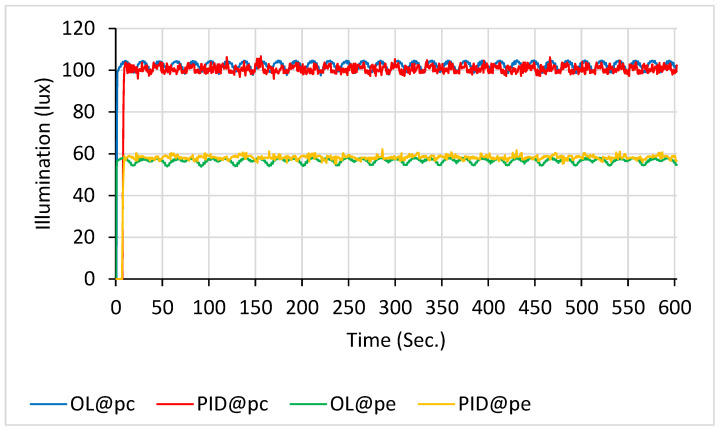
Illuminance with 36 LEDs scanning: PID control (at p_c_ in red line and at p_e_ in orange line) vs. open-loop control (at p_c_ in blue line and at p_e_ in green line).

The illumination levels for 36-LED scanning under OL and PID control at p_c_ and p_e_ are shown in Figure 9. The 10 min averages were as follows: 102.3 ± 1.78 lx (blue, OL@p_c_); 100.6 ± 1.6 lx (red, PID@p_c_); 56.8 ± 1.02 (green, OL@p_e_); and 58.3 ± 0.9 (orange, PID@p_e_).

**Figure 10:**
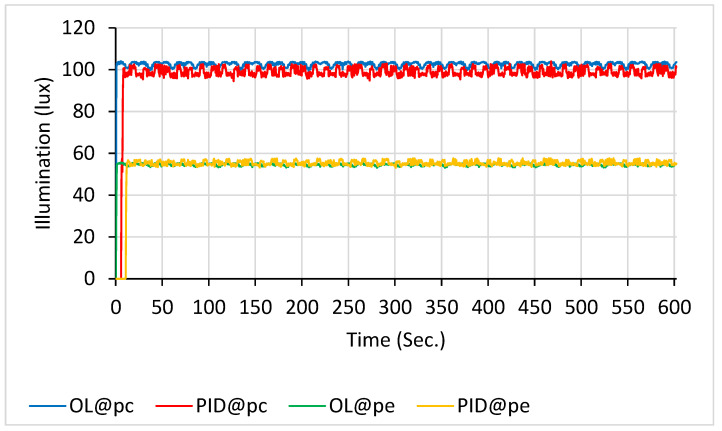
Illuminance with 45 LEDs scanning: PID control (at p_c_ in red line and at p_e_ in orange line) vs. open-loop control (at p_c_ in blue line and at p_e_ in green line).

The illumination results for 45 LEDs scanning in the OL and PID controls at pc and pe are shown in Figure 10. The corresponding 10 min averages were 102.7 ± 1.0 (blue line, OL@pc), 99.0 ± 1.9 (red line, PID@pc), 54.6 ± 0.5 (green line, OL@pe), and 55.3 ± 1.01 (orange line, PID@pe).

## 4. Discussion

The configuration of signal sources within the array structure provides a systematic framework for analyzing the generated fields. Numerous field measurement studies have employed source arrays to achieve time-variant beamforming, utilizing technologies such as laser sources [29], ultrasound [30], and antennas [31]. In line with this approach, our study applied linear system principles to investigate illumination generation and characterize the system characteristics of the incubator. Previous research has also proposed the use of localized and conformal therapies to minimize unnecessary energy exposure. For example, Xiang et al. [32] explored 3D conformal intensity-modulated proton-beam radiation therapy, while Chambers et al. [33] investigated intensity-modulated bolus-electron conformal therapies. Furthermore, Reda and Mohammad’s work [34] bridges the gap between the theoretical advantages of LEDs and the practical requirements of medical device standards, offering a proven solution for creating high-performance phototherapy systems. The study successfully demonstrated that achieving medically compliant irradiance uniformity in LED phototherapy devices is an achievable engineering goal. The key is to optimize the layout of the LED array, specifically the ratio of the mounting height to the LED spacing. In our proposed incubator, a microcontroller was programmed to control the ON/OFF states and illumination levels of the LEDs, thereby enabling conformal therapy by generating passive radiation patterns that reduce ineffective radiation. The scanning modes include electronic and mechanical scans (Supplementary Materials) to meet the uniformity that Reda and Mohammad mentioned.

According to the fundamental principles of optics, the radiation angle of LEDs and their spatial arrangement significantly influence radiation uniformity. Consequently, several studies have focused on optimizing the distribution of light sources to achieve uniform light intensity for NNJ treatment [35]. These investigations have primarily relied on computer simulations and system implementations to achieve global uniformity in phototherapy incubators. However, uniformity across both global and local spaces has not been thoroughly explored. While most studies have focused on radiation uniformity within a 3D incubator space, achieving global radiation uniformity in height does not necessarily ensure uniform radiation across the local surface of the newborn’s skin.

De Gelidi et al. [36] reported that the chest shapes of infants at a mean gestational age of 39 weeks can be approximated as circles with a mean radius of 6.6 cm. Accordingly, in cross-sectional views, both the hands and chest of a newborn were modeled as half-ellipses. Building on this principle, our proposed system can radiate blue light on the newborn’s skin more uniformly, owing to its conformal design to the curvature of the newborn’s chest. The incident blue light is directed perpendicularly onto the skin surface, thereby facilitating greater illumination uniformity than conventional incubator geometries. Furthermore, illumination uniformity can be fine-tuned by adjusting both the duty cycle and the LED module scanning speeds, which are tailored to the specific shape of the newborn’s skin and the distance between the LEDs and skin.

We present computer simulation results along the light intensity contours to validate the advantages of the conformal cover design in achieving local illumination uniformity. All simulations were conducted using MATLAB (version 2021a, MathWorks^TM^, Natick, MA, USA) development environment. Computer simulations were performed on a two-dimensional (2D) plane to simplify the model. In these simulations, the LED light sources were positioned along the inner surface of the cover, which was modeled as an ellipse with major and minor radii of 1.0 and 0.8 m, denoted by a and b, respectively. Ideally, the length of the curved cover with the shape of an ellipse is assumed to be infinite. The light intensity at a specific point (*p*) within the ellipse is inversely proportional to the distance between *p* and each light source. Consequently, the light intensity distribution across a cross-section can be determined. This calculation is analogous to the method employed to evaluate the magnetic field distribution generated by a constant current flowing through an ideal, infinitely long metal wire—a concept widely covered in fundamental physics textbooks.

For the computer simulations, 2001 light sources were employed, each spaced 1 mm apart along the *X*-axis. The simulated intensity at each measurement point was calculated under the condition that all LEDs were activated at a 100% duty cycle, with radiating points also spaced 1 mm apart.

**Figure 11:**
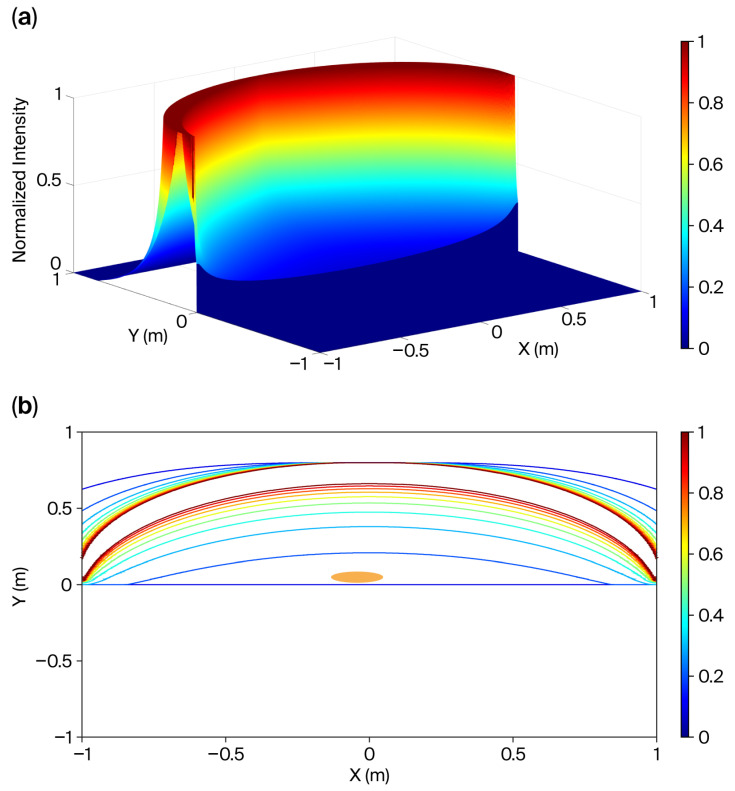
Normalized light intensity distribution. A total of 2001 PWM light sources with 100% duty cycles were used, and the projected distance between one light source and its neighbor on the *X*-axis was 1 mm. (**a**) Three-dimensional (3D) plot; (**b**) the normalized light intensities of the contours are 0.1, 0.2, 0.3…, 1.0. The orange ellipse denotes the cross-section of the newborn.

The normalized illumination distribution is shown in Figure 11. To highlight the intensity range of interest, the illumination peaks were normalized to a value of 1. Accordingly, the contours shown in Figure 11a are further detailed in Figure 11b, with normalized light intensity contours at intervals of 0.1, ranging from 0.1 to 1.0. Assuming the newborn’s cross-sectional shape is elliptical with a = 5 cm and b = 4 cm, the normalized illumination within this region falls between 0.1 and 0.2.

To demonstrate the flexibility of the conformal cover design, we adjusted the intensities of the 2001 light sources according to the following functions:

(9) $$I_{e}\left(x_{e},y_{e}\right)=A\sqrt{\left|x_{e}\right|},$$

where $I_{e}$ denotes the light intensity within the ellipse, which can be controlled via the duty cycle, and A denotes the amplitude of the light source. For computational simplicity, A was set to unity. The position ($x_{e}$, $y_{e}$) represents the coordinates of the light source within the 2D simulation framework.

**Figure 12:**
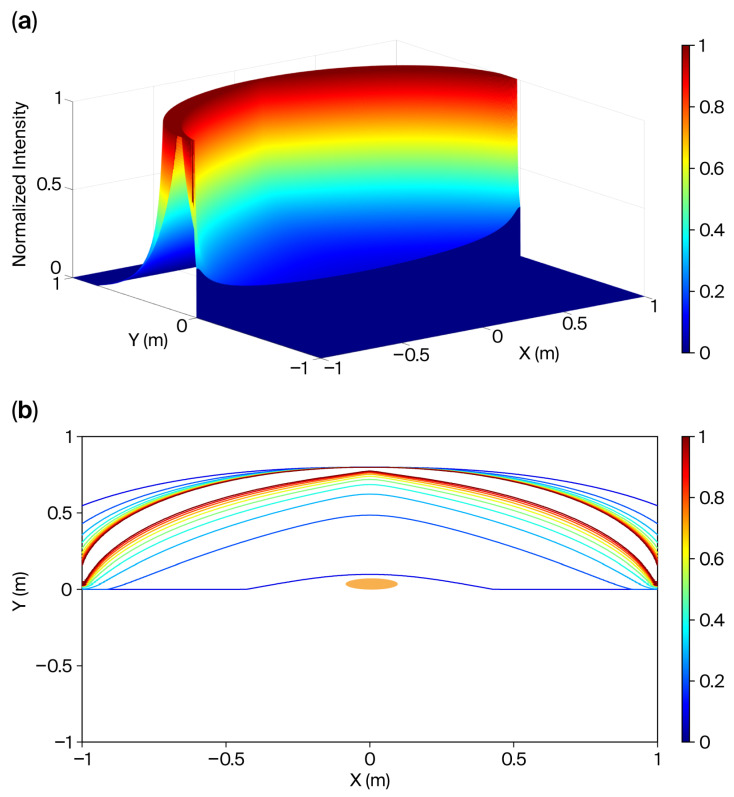
Adjusted normalized light intensity distribution. A total of 2001 PWM light sources, whose intensity was governed by Equation (9), were used, and the projected distance between one light source and its neighbor on the *X*-axis was 1 mm. (**a**) Three-dimensional plot; (**b**) the normalized light intensities of the contours are 0.1, 0.2, 0.3…, 1.0. The orange ellipse denotes the cross-section of the newborn.

The adjusted normalized illumination distribution is shown in Figure 12. To enhance visualization within the targeted intensity range, the illumination peaks were normalized to 1. Consequently, the contours shown in Figure 12a are further detailed in Figure 12b, with normalized light intensity levels at 0.1, 0.2, 0.3…, 1.0. This distribution demonstrated enhanced local uniformity, particularly across the neonatal skin surface.

Notably, the contour shapes varied, reflecting changes in the normalized light intensity, which ranged from 0 to 0.1 on the newborn’s skin. This highlights the advantage of the conformal cover design in achieving local uniformity. Further optimization of contour fine-tuning could be achieved using a pseudo-inverse computation algorithm [37], which is proposed as a direction for future research. In Figure 12, the intensities of the 2001 light sources are adjusted based on the following function:

(10) $$I_{e}\left(x_{e},y_{e}\right)=A\left(1-\sqrt{\left|x_{e}\right|}\right),$$

**Figure 13:**
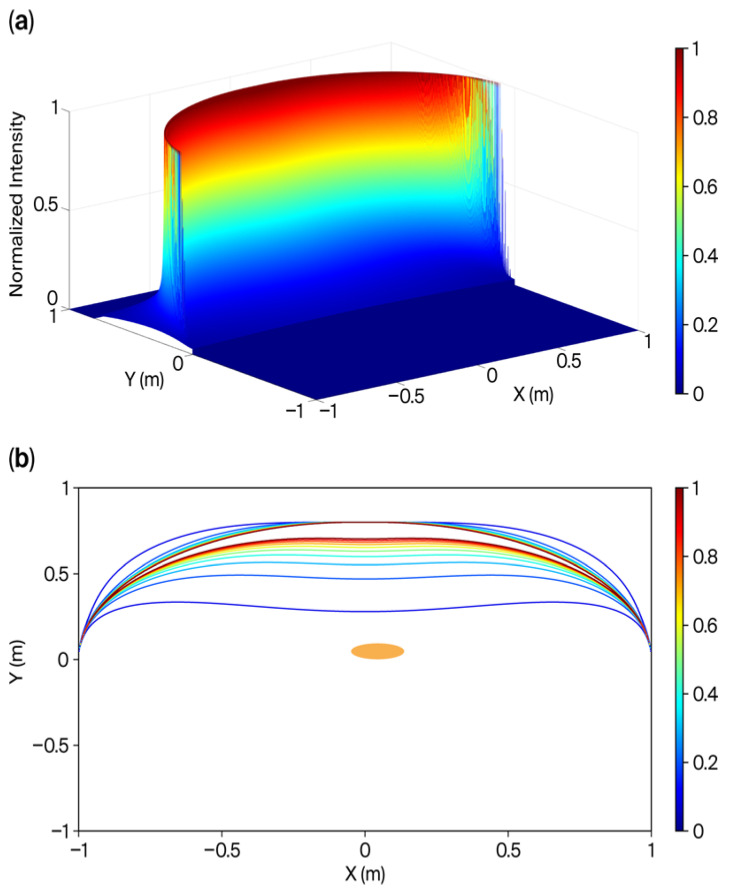
Normalized light intensity distribution. A total of 2001 light sources, with intensities governed by Equation (10), were used, and the projected distance between one light source and its neighbor on the *X*-axis was 1 mm. This demonstrates that the area between 0 and 0.1 of the normalized intensity occupies most of the cross-section. Uniformity is achieved using the method. (**a**) Three-dimensional plot of the distribution; (**b**) respective contours. The orange ellipse denotes the cross-section of the newborn.

The contours in Figure 13a are shown in greater detail in Figure 13b, with normalized intensity intervals of 0.1, 0.2, 0.3, …, 1.0. These results highlight the importance of height uniformity.

Previous studies have primarily focused on contour analysis, as exemplified in Figure 13b, to achieve optimal global light-intensity uniformity for NNJ treatment. The flexibility of the curved cover and LED arrays is also illustrated in Figure 13.

Although this simplified model provides an idealized framework, specific practical considerations should be incorporated in future studies. Notably, this model enables rapid computation of prototyping contours, providing designers with a valuable tool for parameter determination before undertaking more accurate, but computationally intensive, simulations of complex numerical models. To estimate the illuminance on the newborn’s skin surface, a correction factor was applied. The experimental results showed a high degree of consistency between the estimated and actual illuminance values. In confined spaces, computer simulations (Figure 11, Figure 12 and Figure 13) validated that illuminance variation was minimal, suggesting that concerns regarding newborn movement were negligible. Such approximations are generally considered acceptable across multiple research fields.

**Figure 14:**
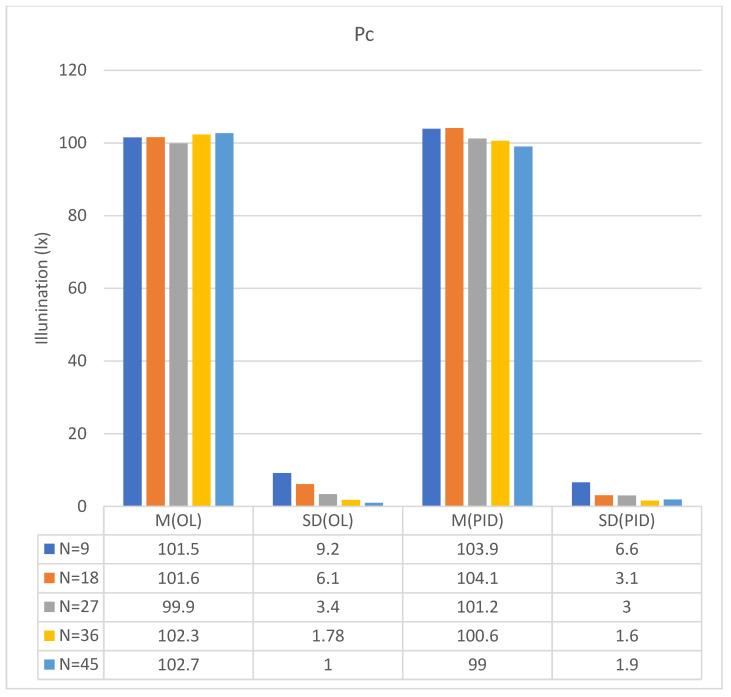
Summary of Figure 6, Figure 7, Figure 8, Figure 9 and Figure 10.

If the electronic scanning speed of the LED array is sufficiently high, the radiating intensity distributions of the array can closely approximate those described by Equations (9) and (10). As a result, the illumination uniformity on the treated newborn’s skin becomes adjustable within the experimental system. Moreover, as previously mentioned, the PID closed-loop control significantly enhanced the illumination stability of the scanning radiating LED array. Based on the data in Figure 6, Figure 7, Figure 8, Figure 9 and Figure 10, the mean and standard deviation of the open-loop control are denoted as M (OL) and SD (OL), respectively, whereas those for the PID closed-loop control are represented as M (PID) and SD (PID), as shown in Figure 14. The findings indicate that the trend of M (PID) approached the PID control set point of 100 lx when N ranged from 9 to 45, in increments of 9. In addition, a comparison of SD (OL) and SD (PID) values clearly confirmed the superior stability achieved by PID control in regulating the radiation output of the scanning LED array. These findings further validate the feasibility of achieving stable illumination contours in computer simulations by employing rapid electronic scanning with a PID control algorithm to modulate the duty cycle.

## 5. Conclusions

This study establishes a refined control framework for illuminating the skin of newborns, representing a significant advancement in the field. Light exposure can be maintained with high stability during high-speed electronic scanning, enabling more precise, reliable, and clinically robust phototherapy. An experimental phototherapy incubator was successfully designed and implemented, incorporating a PID control algorithm for NNJ treatment using blue-light LED arrays with diverse shapes and radiation patterns. The system integrated CFs for real-time monitoring of LED radiation under varying operational conditions. The illumination range of the incubator was sufficient to meet all experimental requirements. By electronically scanning the LEDs, the incubator generated a wide array of phototherapy radiation patterns, thereby supporting the development of conformal therapy techniques. Despite its opaque enclosure structural design, the incubator also enabled effective observations of the internal environment by medical personnel through a dedicated display interface. Additionally, appropriate safety measures were implemented to minimize medical staff exposure to blue-light radiation.

The experimental results indicated that, in most cases, LED radiation exhibited linear regression characteristics. This observation is particularly significant, as it streamlines the design and implementation of linear system control algorithms and reinforces the suitability of this experimental platform for further research. A comparative analysis of SD (OL) and SD (PID) values revealed that the PID control method substantially enhanced stability during the radiation process of the scanning LED array. These results were further supported by computer simulations, indicating that stable output contours could be consistently achieved by integrating rapid electronic scanning with a PID control algorithm tailored to regulate the PWM signals’ duty cycle.

Moreover, light-intensity measurements based on a linear system model validated the feasibility of utilizing blue-light phototherapy in various configurations for the safe treatment of newborns with NNJ. The results also confirmed the successful implementation of indirect light intensity measurements using correction factors across different test cases. Ongoing research is focused on advancing LED-based phototherapy units, such as the system presented in this study, aiming to improve light delivery mechanisms, refine control over light intensity, and optimize spectral composition. These advancements reflect the current trajectory in blue-light phototherapy for NNJ treatment.

From a cost perspective, the incubator used in this study may be somewhat more expensive. However, in most countries, while economic growth is on the rise, the number of annual births has been declining. As a result, we believe many parents in the regions would be willing to invest in higher-quality care, including phototherapy, to ensure the best outcomes for their babies.

## Data Availability

The original contributions presented in this study are included in the article. Further inquiries can be directed to the corresponding author.

## Supplementary Materials

The following supporting information can be downloaded from: Electronic (https://youtu.be/CJlRv9QspfI) and mechanical scans (https://youtu.be/AnVA0o-n5dc) have been available on YouTube since 28 May 2023.

## Abbreviations

The following abbreviations are used in this manuscript:

LED	Light-emitting diode
NNJ	Neonatal jaundice
PWM	Pulse-width modulation
PID	Proportional–integral–derivative

## Appendix A

Based on the CF computing technology described in a previous study [27], the functions of the CFs for N = 9, 18, 27, 36, and 45 are as follows:

**Table A1:** The CF functions of N = 9, 18, 27, 36, and 45.

N	CF_N_
9	${CF}_{9}\left(x\right)=\frac{1.1884x+0.7952}{0.6192x+0.8238}$
18	${\mathrm{CF}}_{18}\left(x\right)=\frac{\;2.5079x+4.8429}{1.3455x+1.5095}$
27	${CF}_{27}\left(x\right)=\frac{3.164x+36.643}{1.5343x+15.262}$
36	${CF}_{36}\left(x\right)=\frac{3.9132x+57.171}{1.9241x+29.029}$
45	${CF}_{45}\left(x\right)=\frac{4.644x+76.31}{2.1789x+79.052}$
